# Extending the Shelf Life of Fresh Khalal Barhi Dates via an Optimized Postharvest Ultrasonic Treatment

**DOI:** 10.3390/plants11152029

**Published:** 2022-08-04

**Authors:** Diaeldin O. Abdelkarim, Isam A. Mohamed Ahmed, Khaled A. Ahmed, Mahmoud Younis, Hany M. Yehia, Assem I. Zein El-Abedein, Abdulla Alhamdan

**Affiliations:** 1 Chair of Dates Industry & Technology, King Saud University, Riyadh 11451, Saudi Arabia; 2 Department of Food Science and Nutrition, College of Food and Agricultural Sciences, King Saud University, Riyadh 11451, Saudi Arabia; 3 Agricultural Research Centre, Agricultural Engineering Research Institute (AEnRI), Giza 12619, Egypt; 4 Department of Food Science and Nutrition, College of Home Economics, Helwan University, Cairo 11611, Egypt; 5 Department of Agricultural Engineering, College of Food and Agricultural Sciences, King Saud University, Riyadh 11451, Saudi Arabia

**Keywords:** total viable count, firmness, total phenolic content, optimization, ultrasonic intensity, response surface methodology

## Abstract

The Barhi date is a high-quality date cultivar whose fruits (dates) are plucked and eaten fresh when they reach the Khalal maturity stage due to their sweetness, crispiness, and yellow skin color. After harvesting, Khalal Barhi fruits rapidly matured to the Rutab stage, where their tissues become soft and their skin color browner. This results in a decrease in their market value and customer demand. This study aims at investigating the effectiveness of the postharvest ultrasonic treatment in conserving the physical, microbial, and nutritional quality of Barhi fruits and extending their shelf life. To achieve the goals of the present work, the response surface methodology (RSM) was used for the optimization of the ultrasonic intensity (50, 100, 150, and 200 W/cm^2^) and application time (5, 10, 15, and 20 min) to preserve the Barhi dates high quality features for varied storage temperatures (1, 5, 15, and 25 °C) and duration (1, 6, 16, and 21 days). In RSM, a four-factors-mixed-levels central composite rotatable design (CCRD) was applied to optimize the ultrasound treatment and storage environments for better-quality physical [total soluble solids (TSS), firmness, and total color changes (ΔE)], microbial [total viable count (TVC)], nutritional [total phenolic content (TPC), DPPH antiradical activity, glucose, and fructose] features of Barhi dates. The outcomes showed that ultrasound intensity and its application time, storage temperature, and storage period influence the physical, microbial, and nutritional quality attributes in different magnitudes. The ideal settings for lessening the changes in the physical attributes, eliminating the microbial growth, and improving the nutritional quality attributes were 140 W/cm^2^, 5.2 min, 20.9 °C, and 21 days for ultrasound intensity, ultrasound exposure duration, storage temperature, and storage duration, respectively. In conclusion, this study proved the potential application of ultrasound for persevering the excellence aspects of Barhi dates and identified the ideal ultrasound environments for maintaining the physical, microbial, and nutritional quality features of Barhi dates during extended storing.

## 1. Introduction

The date palm (*Phoenix dactylifera* L.) fruits (dates) are a main basis of food in North African and Arabic countries, where numerous date cultivars are planted and play an important portion in the society culture, history, and economy [1]. The dates are non-climacteric fruits, so they have very little metabolic activity [2]. Dates contain high levels of sugars, phenols, flavonoids, carotenoids, anthocyanin, vitamins, and minerals, as well high levels of soluble fiber and low-fat content, making them nutritious and offering quick energy intake with some therapeutic, antioxidant, and anti-mutagenic properties effects [3,4,5,6].

**Figure 1:**
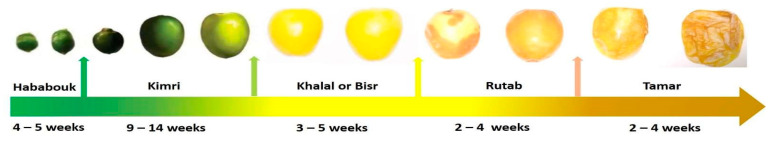
Different growth and maturity stages of Barhi date fruit (post-anthesis weeks).

There are five stages of dates maturation and ripening viz. Hababouk, Kimri, Khalal (Bisr), Rutab, and Tamer (Figure 1). Dates are usually picked and sold in the third, fourth, and fifth maturity stage and may be eaten soft, semi-dry, or dry, subject to the variety characteristics, climate, and market demand [1]. The Barhi date cultivar, a popular date variety extensively farmed in the Middle East [7], is often plucked, and consumed fresh during the Khalal stage of maturity, as the fruits are crunchy and delicious with an appealing yellow skin color [8]. After harvesting, Barhi fruits quickly change to the Rutab stage while their tissues soften and the skin color darkens; nevertheless, consumer demand and market prices plummet [9]. Therefore, the slowing down of the fruit’s ripening is a significant necessity for marketing harvested Khalal Barhi dates.

Ultrasound is regarded as an innovative and appealing technology in the food sector, since it offers a distinct advantage over other technologies. Acoustic waves produced by ultrasound power are deemed safe, non-toxic, and ecologically benign [10]. Abundant reports have been published about the impact of ultrasound treatment on food handling and maintenance [11,12,13]. Nonetheless, many of these reports have concentrated on deactivating microbes and enzymes, extracting antioxidant chemicals, and speeding up heat transmission [11]. To date, information regarding the impact of ultrasound action on the preservation of Barhi dates quality is limited. Therefore, the purpose of this research was the optimization of the ultrasonic treatment of Barhi dates in terms of ultrasound intensity and application duration, as well as to evaluate the influence on deterioration rate, microbial count, and quality preservation in optimal cold storage situations.

## 2. Results and Discussion

### 2.1. Model Fitting

Postharvest processing circumstances are very important issues that affect the storability and quality attributes of Barhi dates, which are mostly traded and consumed at a very perishable Khalal maturity stage [14]. At this stage, the freshness and shelf life of Barhi dates are greatly influenced by improper postharvest processing conditions, which might accelerate the senescence, ripening, respiration, and microbial spoilage processes [15]. To overcome such problems, the present study was conducted to evaluate the influence of ultrasound usage and subsequent storage circumstances on the physicochemical quality aspects of Barhi dates and to optimize the ultrasound usage and storage environments using the response surface methodology (RSM) model. The RSM model’s adequacy and precision were validated by adequacy precision and coefficient of determination indicators (CV, R^2^ and adjusted R^2^) as displayed in ANOVA analysis (Table 1). The intercept of RSM model was significant (*p* < 0.05) for TSS, TPC, and fructose; highly significant (*p* < 0.01) for firmness, TVC, DPPH, and glucose; and extremely significant for ∆E, indicating good fitting of the model for all assessed attributes. In addition, the lack of fit was insignificant (*p* > 0.05) of all assessed attributes suggesting the used models adequately described the experimental data. The high coefficients of determination (R^2^) were observed for TSS, firmness, ∆E, TVC, TPC, DPPH, glucose, and fructose, which were ranged from 0.925 to 0.992, suggesting that the applied quadratic polynomial models (Equation (9)) contributed to more than 92% of the total variability of traits. Similar observations were recently reported for Barhi dates treated with infrared at different doses and duration and storage at different temperature and times [15]. Comparable results of coefficients of determination (R^2^), predicted R^2^ and adjusted R^2^ indicate a good statistical model [16]. The current results showed that adjusted R^2^ of the assessed attributes were in the range of 0.871 to 0.958, which was comparable to that of R^2^ (0.925 to 0.992) indicating high significance of the applied model that is similar to our recent observations on RSM model used for optimizing infrared treatment of Barhi dates [15]. In addition, the coefficient results also indicated high correlations between predicted and actual values of TPC, TSS, firmness, DPPH, ∆E, TVC, glucose, and fructose because the coefficient values (R^2^ and adjusted R^2^) were near to one [16,17]. Adequacy precision indicates the ratio of signal-to-noise and which high ratio (>4) is desirable and indicates good model fitting [16,18]. In this regard, the adequacy precision of the assessed attributes were in the array of 7.913 to 16.535, demonstrating that the used model is adequate to analyze the data. Another indicator for reproducibility and precision of the models is the coefficient of variation (CV) where low CV (<5.0%) indicates high reproducibility and precision of the used models [16,18]. The CV of TSS, firmness, ∆E, TPC, DPPH, TVC, glucose, and fructose were lower (1.088–3.731%) than 5.0% suggesting great precision and reproducibility of the data and great fitting of the used RSM model. Based on the aforementioned discussion, experimental data in this study are precise and reproducible and the chosen RSM models are appropriate for optimizing the ultrasound conditions and subsequent storage settings for extending the shelf life of Barhi dates while retaining its physicochemical and nutritional quality attributes.

### 2.2. Effect of US Treatment and Storage Environments on Physical Properties of Barhi Dates

The results on the effect of independent variables (US intensity, US exposure duration, storage temperature, and storage duration) on the physical attributes (TSS, firmness, and ∆E) of Barhi dates and their multiple regression analysis coefficients are shown in Table 1. The RSM models used were significant (*p* < 0.05), highly significant (*p* < 0.01), and extremely significant (*p* < 0.001) for TSS, firmness, and ∆E, respectively suggesting good fitting of the selected RSM models. In linear terms, US intensity had a positive (*p* < 0.05) influence on the TSS suggesting that increasing US intensity would increase the TSS of Barhi dates, whereas storage duration had a negative (*p* < 0.01) effect on the TSS, suggesting that elongation of storage time might reduce the TSS of Barhi dates. The interaction effect of US intensity, US duration, US intensity, and storage time showed positive (*p* < 0.05; *p* < 0.01) effects on the TSS indicating that combinations of increased US intensity with duration and increased US intensity with storage duration could increase the TSS of Barhi dates. The rise of TSS after the increase in US intensity and duration is prospectively due to a decrease in moisture content and an increase in respiration and enzymatic processes, which lead to the transformation of non-reducing sugars into reducing sugars, thereby increasing the TSS of Barhi dates [19]. The reduction in TSS during prolonged storage is likely due to the senescence, which lead to degradation and/or reduction in TSS in fruits and vegetables [20]. Similarly, previous reports have shown that ultrasound treatment increased the TSS of cherry [19] and mango [21]. In addition, reduction in TSS during storage was reported in ultrasound treated cucumber [20] and green asparagus [22].

In linear terms, US intensity had a greatly negative (*p* < 0.01) influence on the firmness of Barhi dates, indicating that increasing US intensity could reduce the firmness of Barhi dates, whereas storage time had an extremely positive (*p* < 0.001) impact on the firmness suggesting that elongation of storage time could increase the firmness of Barhi dates. In interaction terms, US intensity and storage temperature had a negative impact on the firmness, whereas US exposure duration and storage temperature had a positive influence on the firmness of Barhi dates (*p* < 0.05). The reduction in Barhi date firmness following increased US intensity and storage temperature is likely due to the degradation of cell wall structure by ultrasound waves and high temperature, thereby leading the fruits to be softer. Similar observations on the reduction in kiwi fruit firmness following increased US intensity and temperature and attributed that to the mechanical effects of ultrasound [23]. The increase in firmness following increased storage temperature and US treatment time could be attributed to the evaporation of moisture content from the surface layers of the dates [15,24].

The total color changes (∆E) were highly influenced by the US treatment and storage conditions under linear, quadratic, and interaction terms. In linear terms, US intensity had a negative (*p* < 0.001) effect on the ∆E, while storage temperature and period had positive (*p* < 0.01) effects on the ∆E of Barhi dates. The interactive effects of US intensity and storing temperature, US intensity and storage time, US exposure duration and storage period, and storing temperature and storing duration on ∆E were positive (*p* < 0.01), proposing that an upsurge in these variables would rise the ∆E of Barhi dates. The interactive influence of US exposure duration and storing temperature on ∆E was negative (*p* < 0.01), indicating that increase in these variables would decrease the ∆E of Barhi dates. In the quadratic terms, US intensity had a positive effect (*p* < 0.05) on ∆E, indicating that an increase in US intensity would increase the ∆E of Barhi dates, while storing temperature had a negative (*p* < 0.05) effect on the ∆E, demonstrating that increasing the storage temperature would reduce the ∆E of Barhi dates. The increment of ∆E values following US usage and storing environments could be due to sonochemical reaction effects caused by ultrasound, which affect the oxidative stability of sensitive colorants, such as chlorophyll and carotenoids, thereby leading to color decomposition and degradation [19,25]. The reduction in ∆E values following increased US intensity is likely due to the cavitation effects of ultrasound that cause reductions in the metabolic rate, thereby preserving the color of Barhi dates [20]. Similar observations on the impacts of ultrasound usage and storing period on the total color changes (∆E) of various fruits and vegetables have been reported [19,20,25]. The prediction equations specifying the effects of US usage and storing settings on the physical quality attributes (hardness, TSS, and ∆E) of Barhi dates using significant terms are as follows:

(1) $$Y_{TSS}=27.677+0.677X_{1}-2.513X_{4}+0.016X_{1}X_{2}+0.020X_{1}X_{4}$$

(2) $$Y_{hardness}=332.997-7.010X_{1}+18.583X_{4}-0.100X_{1}X_{3}+0.085X_{2}X_{3}$$

(3) $$Y_{\Delta E}=156.733-8.115X_{1}+5.038X_{3}+27.525X_{4}+0.058X_{1}X_{3}+0.063X_{1}X_{4}-0.154X_{2}X_{3}+0.079X_{2}X_{4}+0.075X_{3}X_{4}+0.033X_{1}^{2}-0.179X_{3}^{2}$$

**Figure 2:**
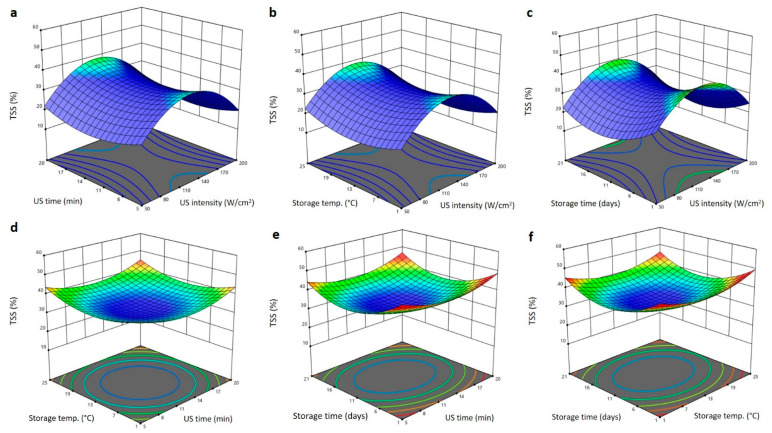
Response surface plots of total soluble solids (TSS) of Barhi dates as a function of US time and US intensity (**a**), storage temperature and US intensity (**b**), storage time and US intensity (**c**), storage temperature and US time (**d**), storage time and US time (**e**), and storage time and storage temperature (**f**).

**Figure 3:**
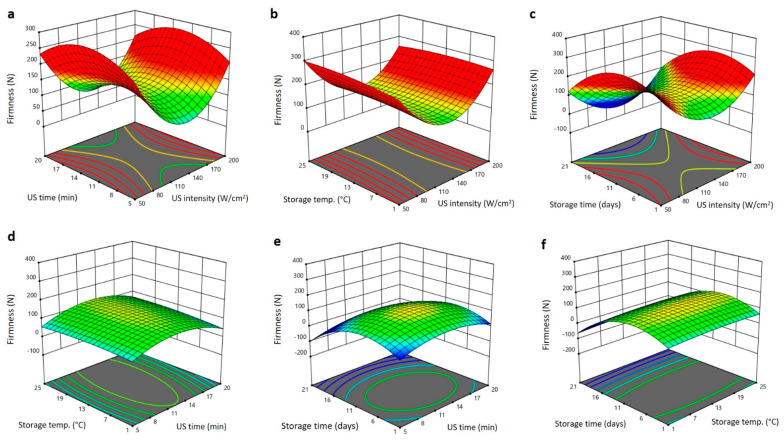
Response surface plots of firmness of Barhi dates as a function of US time and US intensity (**a**), storage temperature and US intensity (**b**), storage time and US intensity (**c**), storage temperature and US time (**d**), storage time and US time (**e**), and storage time and storage temperature (**f**).

**Figure 4:**
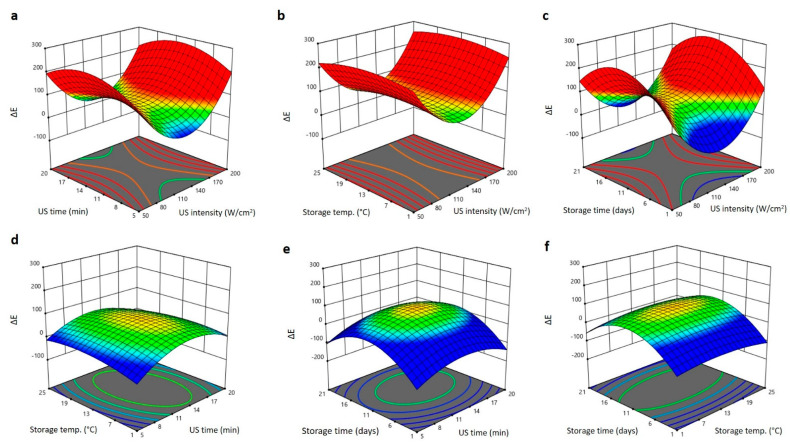
Response surface plots of total color changes (ΔE) of Barhi dates as a function of US time and US intensity (**a**), storage temperature and US intensity (**b**), storage time and US intensity (**c**), storage temperature and US time (**d**), storage time and US time (**e**), and storage time and storage temperature (**f**).

To determine the interactive effects of independent variables on the physical characteristics (TSS, firmness, and ∆E) of Barhi dates and, subsequently, approve the ideal levels of individual variable for that high reaction, three dimensional (3D) surface blots were created (Figure 2, Figure 3 and Figure 4). At fixed US exposure duration, storing temperature, and storing period, increasing US intensity augmented the TSS of Barhi dates to the highest values at 140 W/cm^2^ and then reduced again at high US intensity (Figure 2a–c). Upsurge in the US exposure duration, storing temperature, and storing period reduced the TSS of Barhi dates to the least values at 11 min, 13 °C, and 11 days, respectively. It then augmented again to the highest levels as the US time, storing temperature, and storing duration elongated to 20 min, 25 °C, and 21 days, respectively (Figure 2a–f). The high TSS of Barhi dates at US intensity of 140 W/cm^2^ could be due to the enhancement of respiration and enzymatic metabolic processes that cause the conversion of polysaccharides into reducing sugars and releasing [19], however, high US intensity (>140 W/cm^2^) might inhibit the metabolic enzymes and hence reduced TSS of Barhi dates. In addition, increased US treatment time and storage temperature, in addition to US intensity, might disrupt the cellular matrix and released more soluble components and thereby augmented the TSS of Barhi dates [21].

As the US intensity augmented, the firmness of Barhi dates reduced to the minimum values at 140 W/cm^2^ and then augmented again to the highest levels as the US intensity raised to 200 W/cm^2^ (Figure 3a–c). Upsurge in the US exposure duration and storing period augmented the firmness to the maximum values at 14 min and 11 days and then declined again as the US exposure time and storing duration elongated to 20 min and 21 days, respectively (Figure 3a,c–f). Increase in the storing temperature gradually augmented the firmness of Barhi dates to the highest levels at 25 °C (Figure 3b,d,f). The decline of firmness of Barhi dates at US intensity of 140 W/cm^2^ and longer storage temperature is likely due to increased activities of metabolic enzymes, which accelerate the ripening process and thereby increases the softening of Barhi dates [26]. In addition, prolonged exposure of Barhi dates to US could also induce cavitation and affect the cell wall stability, thereby leading to a reduction in firmness [20]. The increase in firmness following the increase in US treatment and storing environments is possibly owing to the evaporation of water from the surface of Barhi dates making the surface harder and firmer [15,24].

The total color changes (ΔE) were also affected by the US treatment and storage conditions in different manners (Figure 4a–f). Increasing US intensity reduced ΔE to the minimum values at 140 W/cm^2^,whereas further increase in US intensity increased the ΔE to maximum values ate 200 W/cm^2^ (Figure 4a–c). Increasing the US time and storage time increased the ΔE to the maximum values at 14 min and 15 days, however, further increases in the US treatment and storing duration reduced ΔE of Barhi dates (Figure 4a,c–f). Increasing the storage temperature gradually increased the ΔE of Barhi dates reaching their highest value at 18 °C, and thereafter slightly declined at a high temperature of 25 °C (Figure 3b,d,f). The high of ΔE following the treatment of Barhi dates at high US intensity (200 W/cm^2^) and moderate US treatment time and storage temperature and duration is probably due to sonochemical reactions and temperature degradation of sensitive pigments [19,25]. Maintaining low ∆E of Barhi dates at moderate US intensity (140 W/cm^2^), short exposure and subsequent storage of the dates at low temperature conditions for up to 21 days is preferable and the results are comparable to previous reports [15,20].

### 2.3. Effect of US Treatment and Storing Conditions on Total Viable Count (TVC) of Barhi Dates

The results in Table 1 show that the US treatment and storing environments possessed significant effects on the TVC of Barhi dates. In linear terms, US intensity had a negative effect (*p* < 0.01) on the TVC of Barhi dates, indicating that an increase in the US intensity would decrease the TVC of Barhi dates. Storage temperature and time had positive (*p* < 0.05) effects on the TVC of Barhi dates proposing the elevating storing temperature and storage duration would increase the TVC of the product, and hence reduce its shelf life. In interaction terms, US intensity and US time, US intensity and storing period, and US exposure duration and storing duration had positive effects on the TVC, whereas US exposure time and storing temperature had a negative influence on the TVC of Barhi dates. In quadratic term, storing period had a negative effect on the TVC of Barhi dates. The reduction in TVC following US treatment is likely due to the cytolytic effects that are caused by ultrasound, which lead the inactivation of microbes [27]. Increase in the TVC during elongated storing of Barhi dates is expectedly due to increased TSS and reducing sugars that enhance the growth of microorganism by acting as rapid and easy carbon sources for microorganisms [15]. The prediction equation for specifying the influence of US usage and storing duration on the TVC of Barhi dates using significant terms is as follows:

(4) $$Y_{TVC}=32.134-1.258X_{1}+1.381X_{3}+4.476X_{4}+0.015X_{1}X_{2}+0.012X_{1}X_{4}-0.024X_{2}X_{3}+0.011X_{2}X_{4}-0.209X_{4}^{2}$$

**Figure 5:**
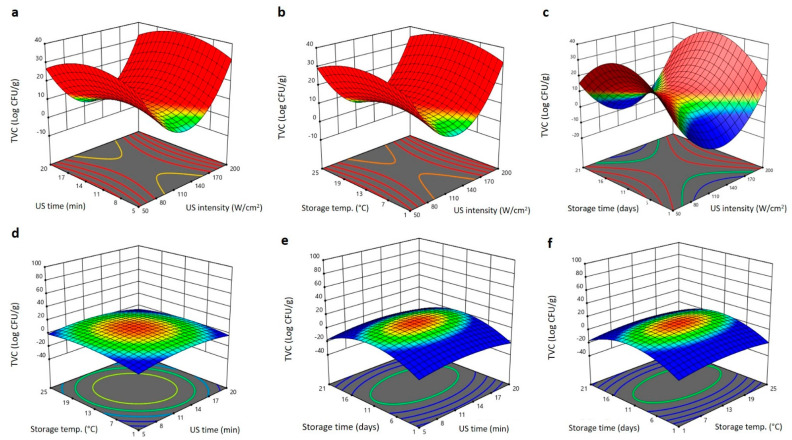
Response surface plots of total viable count (TVC) of Barhi dates as a function of US time and US intensity (**a**), storage temperature and US intensity (**b**), storage time and US intensity (**c**), storage temperature and US time (**d**), storage time and US time (**e**), and storage time and storage temperature (**f**).

The 3D surface plot analysis showed that TVC of Barhi dates was affected by the US treatment and storage conditions in different manners (Figure 5a–f). Increasing US intensity reduced the TVC to the minimum values at 130–140 W/cm^2^ and then increased again to the same initial levels as intensity elevated to 200 W/cm^2^ (Figure 5a–c). Upsurge in the US exposure duration, storing temperature, and storing duration augmented the TVC to highest levels at 14 min, 17–19 °C, and 13–15 days and thereafter declined again as these variables augmented to 20 min, 25 °C, and 21 days, respectively (Figure 5a–f). These outcomes specify that application of moderate US intensity (130–140 W/cm^2^) for short duration is suitable for reducing the TVC of Barhi dates during storage at moderate temperatures for up to 21 days. The low TVC of Barhi dates treated with moderate US intensity (130–140 W/cm^2^) is likely due to the formation of large cavitation bubbles by moderate ultrasound frequency, which lead to the generation of high temperature and pressure in the cavitation region, thereby increasing the disruption rate of microbial cells and then causing the inactivation of microbes [20]. In addition, the prolonged exposure of Barhi dates to high US intensity (200 W/cm^2^) increased the TVC due to cell wall damage caused by generated temperature and pressure by ultrasound, which permit the penetration of microbes to the inside of the fruit, and thus increase the TVC of the samples [19]. Similar observations on the influence of US treatment and storing conditions have been reported for cherry [19], fresh-cut cucumber [20], and strawberries [28].

### 2.4. Effect of US Treatment and Storing Conditions on Bioactive Features of Barhi Dates

Bioactive compounds, such as phenolic and flavonoid compounds, are found in ample quantities in date fruits, and these compounds are known for their nutritional and health potentials as antioxidant, antimicrobial, antihyperlipidemia, antidiabetic, and anticancer components [7]. In this work, US treatment and storing settings were found to exhibit variable effects on the total phenolic content (TPC) and DPPH antiradical activity of Barhi dates (Table 1). In linear terms, regression analysis displayed significantly (*p* < 0.01) negative effects of storing temperature and storage duration on the TPC, demonstrating that increasing the storing temperature and elongating the storing period would reduce the TPC of Barhi dates. In quadratic terms, US intensity possessed negative (*p* < 0.01) effects on the TPC suggesting a reduction in TPC as US intensity increased, whereas US exposure duration, storing temperature, and storing period displayed positive (*p* < 0.01) effects on the TPC, indicating increases in these variables would enhance the TPC of Barhi dates. The decrease in TPC of Barhi dates following the increase in US intensity is probably due the degradation of phenolic compounds by excessive cavitation, cell disruption, oxidation reactions, and reducing extractability of phenolic compounds [15,19,21,25]. The increase in TPC following the increase in storage temperature and period is mostly due to the enzymatic liberation of bound phenolic compounds from the cellular matrix, changing structure, and increasing the solubility and extractability of phenolic compounds [15,19].

Regression analysis also showed variable (*p* < 0.01) impacts of US settings and storing conditions on the DPPH antiradical activity (Table 1). In linear terms, US intensity and storage temperature exhibit positive (*p* < 0.01) effects on the DPPH antiradical activity revealing that increasing US intensity and storage temperature will increase the DPPH antiradical activity of Barhi dates. Storage period exhibited (*p* < 0.01) negative influence on the DPPH antiradical activity, indicating that elongating storage would decrease the DPPH antiradical activity of Barhi dates. In the interaction term, a combination of US intensity and storing temperature resulted in a reduction in DPPH antiradical activity as the negative (*p* < 0.01) effect of these variables on DPPH antiradical activity of Barhi dates was observed. The increase in DPPH antiradical activity following the increase in US intensity and storage temperature is probably due the thermal generation of hydroxyl radicals by high ultrasound intensity and temperature [19,29]. In addition, the release of more pound phenolic compounds could also lead to increased DPPH antiradical activity of Barhi dates. The reduction in DPPH antiradical activity by the combination of US intensity and storage temperature, and prolonged storage time might be accredited to the thermal degradation of sensitive phenolic compounds [15,19,21,22,25]. The prediction equations showing the impact of US settings and storing environments on the bioactive properties of Barhi dates are as follows:

(5) $$Y_{TPC}=4.962-25.005X_{3}-34.684X_{4}-0.041X_{1}^{2}+1.248X_{2}^{2}+0.619X_{3}^{2}+1.256X_{4}^{2}$$

(6) $$Y_{DPPH}=63.455+0.660X_{1}+0.693X_{3}-1.611X_{4}-0.028X_{1}X_{3}$$

**Figure 6:**
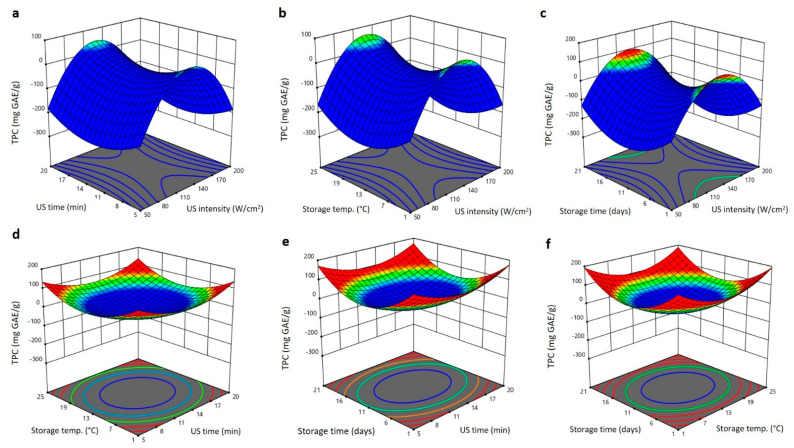
Response surface plots of total phenolic content (TPC) of Barhi dates as a function of US time and US intensity (**a**), storage temperature and US intensity (**b**), storage time and US intensity (**c**), storage temperature and US time (**d**), storage time and US time (**e**), and storage time and storage temperature (**f**).

**Figure 7:**
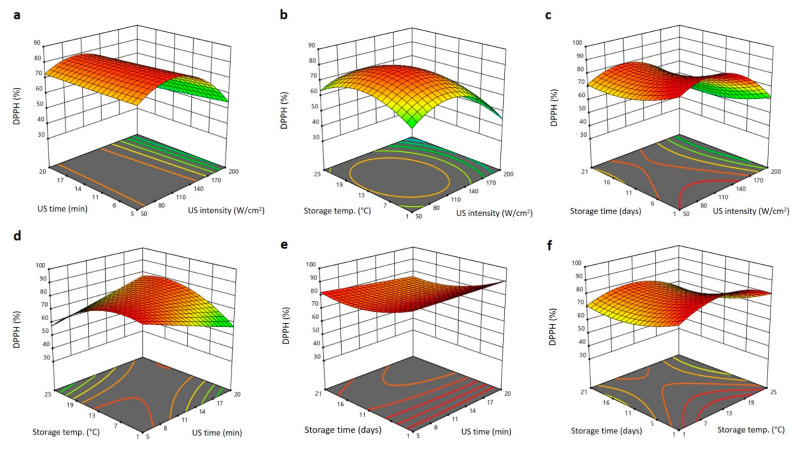
Response surface plots of DPPH antiradical activity of Barhi dates as a function of US time and US intensity (**a**), storage temperature and US intensity (**b**), storage time and US intensity (**c**), storage temperature and US time (**d**), storage time and US time (**e**), and storage time and storage temperature (**f**).

Bioactive properties (TPC and DPPH antiradical) were influenced by US treatment and storage conditions in different manners, as shown in the 3D surface plots (Figure 6 and Figure 7). Increasing the US intensity increased the TPC to maximum levels at 140 W/cm^2^ and then reduced again as the US intensity elevated to 200 W/cm^2^ (Figure 6a–c). Increasing the US time, storing temperature, and storing period declined the TPC of Barhi dates to least levels at 11 min, 13 °C, and 11 days, which were thereafter augmented again to the same initial levels as these treatment variables increased to 20 min, 25 °C, and 21 days, respectively (Figure 6a–f). These outcomes suggest that application of moderate US intensity (140 W/cm^2^) for short duration as postharvest treatment of Barhi dates could increase the shelf life of product for 21 days under both cold (1 °C) and room temperature (25 °C) storage conditions. The upsurge in TPC following US usage is prospectively due to the cleavage of covalent bonds between phenolic compounds and cell wall matrix and thereby increases the liberation of free phenolic compounds. However, at higher US intensity and longer exposure polymerization and degradation, phenolic compounds might occur, leading to the decline of TPC of Barhi dates [15,19,21,22,25].

The 3D surface blots of DPPH antiradical activity of Barhi dates as affected by US treatment and storage conditions are shown in Figure 7a–f. Increasing the US intensity augmented the DPPH antiradical activity to the highest values at 120–130 W/cm^2^ and then reduced to minimum values high US intensity (Figure 7a–c). Storage temperature showed the same trend as US intensity, in which increasing storing temperature augmented the DPPH antiradical activity of Barhi dates to the highest level at 10–13 °C and thereafter declined again as the storing temperature elevated (Figure 7b,d,f). US duration affected the DPPH antiradical activity in varied manner as it showed a gradual increase with an increase in US time at constant US intensity (Figure 7a) and storage time (Figure 7e), whereas it showed gradual reduction increase with increase in US time at constant storage temperature (Figure 7d). The elongation of the storage time slightly reduced DPPH antiradical activity of Barhi dates to the minimum values at 16 days and thereafter marginally increased at the end of storage period (Figure 7c,e,f). The increasing trend in DPPH antiradical activity following postharvest treatment of Barhi dates with moderate US intensity is similar to the increased phenolic compound, suggesting well correlation between TPC and DPPH antiradical activity. In addition, the generation of hydroxyl radicals and releasing of more bound phenolic compounds by high ultrasound and temperature are likely the reasons for improved DPPH antiradical activity of US-treated Barhi dates [19,29].

### 2.5. Effect of US Treatment and Storing Conditions on Glucose and Fructose Content of Barhi Dates

Regression analysis indicated that US usage and storing settings influenced the glucose and fructose content in similar manners (Table 1). In linear terms, US time and storage temperature negatively (*p* < 0.05) affected the glucose and fructose content, suggesting that increasing US time and storage temperature would reduce the glucose and fructose levels of Barhi dates. The decrease in glucose and fructose during the elongated exposure of Barhi dates to ultrasound waves is probably due to generated heat and pressure in the ultrasound liquid at the cavitation region, which lead to the cleavage of glucosidic bonds of polysaccharides and acceleration monosaccharides degradation [15,30]. The prediction equations showing the significant impacts of US usage and storing settings on glucose and fructose are as follows:

(7) $$Y_{Glucose}=14.580-5.101X_{2}-1.634X_{3}$$

(8) $$Y_{Fructose}=24.782-5.826X_{2}-2.312X_{3}$$

**Figure 8:**
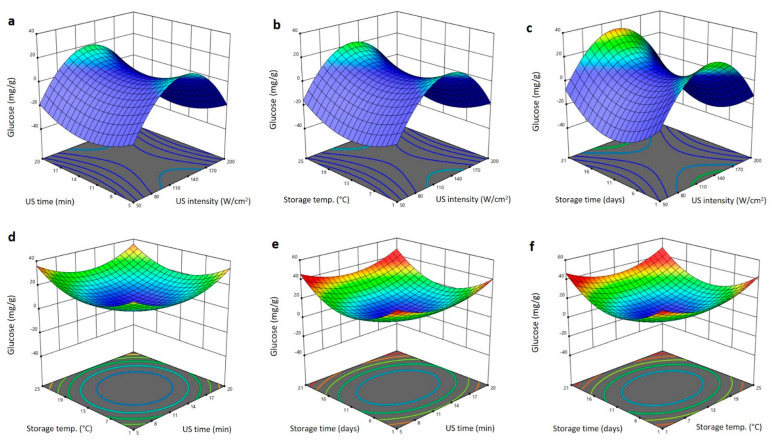
Response surface plots of glucose content of Barhi dates as a function of US time and US intensity (**a**), storage temperature and US intensity (**b**), storage time and US intensity (**c**), storage temperature and US time (**d**), storage time and US time (**e**), and storage time and storage temperature (**f**).

**Figure 9:**
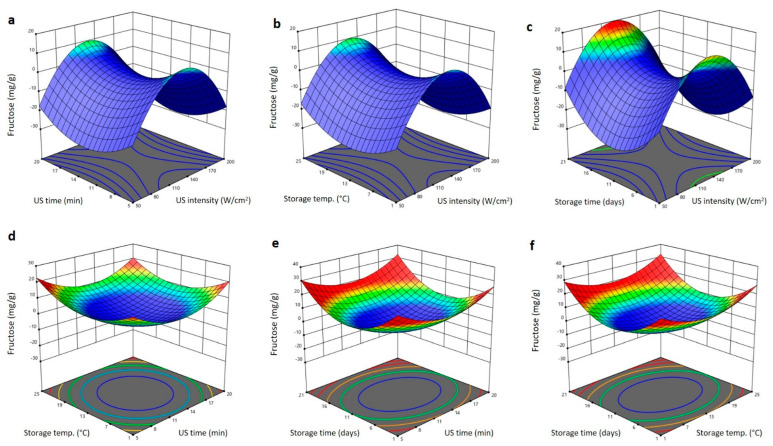
Response surface plots of fructose content of Barhi dates as a function of US time and US intensity (**a**), storage temperature and US intensity (**b**), storage time and US intensity (**c**), storage temperature and US time (**d**), storage time and US time (**e**), and storage time and storage temperature (**f**).

The 3D surface blots showing the impact of US treatment and storage conditions variables on glucose and fructose content are shown in Figure 8 and Figure 9. The increase in US intensity increased the glucose and fructose content to the maximum values at 140 W/cm^2^ and then reduced again as the intensity increased (Figure 8a–c and Figure 9a–c). At fixed US intensity, the upsurge in US duration and storing period declined the glucose and fructose to minimum values at 11 min and 11 days, respectively (Figure 8a–c and Figure 9a–c). The interaction and quadratic terms showed high values of glucose and fructose at extremes of US time (5 and 20 min), storing temperature (1 and 25 °C), and storing duration (1 and 21 days), with the highest values being observed at high US time (20 min), storage temperature (25 °C) and storing time (21 days). However, the lowest values of glucose and fructose being seen in middle range of the US time (12.5 min), storage temperature (13 °C), and storage time (11 days) (Figure 8d–f and Figure 9d–f). Increase in the glucose and fructose in Barhi dates treated at moderate US and storing settings is likely due to an increase in respiration and enzymatic processes that lead to the conversion of polysaccharides and disaccharides into reducing sugars [19,26,31].

## 3. Materials and Methods

### 3.1. Barhi Date Fruits

Barhi dates are preferably harvested and eaten at the Khalal maturity stage [7] and, consequently, fresh Barhi dates at this stage were purchased from a Barhi date retailer in Riyadh City, Saudi Arabia in September 2020. The fruits with the same size, color, maturity stage, and consistent appearance were transferred under cool conditions to the Laboratory, where they were cleaned with compressed air to remove dusts and forging materials [8]. After that, the samples were divided into equal portions and were then immediately subjected to ultrasound treatment and subsequent storage and analysis. The original moisture content and total soluble solids of the samples were 74.33 ± 0.74% and 14.7 ± 0.6%, respectively. The chemical used were of analytical reagent grades and were bought from Sigma Aldrich (Sigma, St. Louis, MO, USA).

### 3.2. Ultrasonic Treatment

Using a probe type ultrasonic generator (Shenzhen Ours Ultrasonic Co. Ltd., Shenzhen, China), fresh Barhi date samples (3 kg per treatment, total 48 kg) were treated with ultrasonic waves at different frequencies (50, 100, 150, 200 kHz) for varying treatment times (5, 10, 15, 20 min). According to the experimental design, the treated Barhi fruits were divided into 0.3 kg portions (25 ± 5 date fruits per portion) and then kept in different type of packs (closed, open, or perforated) and kept at different temperatures (1, 5, 15, and 25 °C) for variable intervals (1, 6, 11, 16, and 21 days). At the beginning of storage and 5 days intervals, total soluble solids (TSS), firmness, total color difference (∆E), total phenolic content (TPC), DPPH antiradical activity, total viable count (TVC), and glucose and fructose content were assessed for Barhi dates.

### 3.3. Designing Experiment

A response surface methodology (RSM) model with a mixed-level four-factor central composite rotatable design (CCRD) was applied for optimization of the ultrasound (intensity and duration) and storage (temperature and duration) settings on the microbial, physical, and nutritional quality attributes of Barhi dates [15]. For RSM model construction and data analysis, a Design Expert software version 11.0 (Stat-Ease Inc., Minneapolis, MN, USA) was applied. In the CCRD, a total of 30 experimental runs containing 6 replicates at the center point were applied. Ultrasound frequencies (X1) (50, 100, 150, 200 kHz), ultrasound treatment duration (X2) (5, 10, 15, 20 min), storage temperature (X3) (1, 5, 15, and 25 °C), and duration (X4) (1, 6, 11, 16, and 21 days) were considered as independent variables (Table 2), whereas TSS, firmness, ∆E, TPC, DPPH, TVC, glucose, and fructose were designed as responses. The expression of the dependent responses as a function of independent factors was designed using the following second-order polynomial equation:

(9) $$Y=\sum \;\beta_{0}+\sum \;\beta_{i}\;X_{i}+\sum \;\beta_{\mathrm{ii}}\;X_{i}^{2}+\sum \;\sum \;\beta_{\mathrm{ij}}\;X_{i}{\;X}_{j}$$

In the above equation, the predicted response, intercept, and regression coefficients of linear, quadratic, and interaction terms are designed as Y, β0, βi, βjj, and βij, respectively, whereas the coded independent variables are Xi and Xj. F test was used for interpretation of coefficients, whereas regression, ANOVA, and surface plotting were conducted to specify the ideal conditions for ultrasound and storage environments of Barhi dates.

### 3.4. Determination of Quality Features of Barhi Dates

The determination of TSS, firmness, ∆E, TPC, DPPH, TVC, glucose, and fructose of the Barhi fruits were done at the first day of storage and during intervals of 5 days during storage at different temperatures.

#### 3.4.1. Determination of TSS

An ABBA5 refractometer (BS instruments, Jena, Germany) was used for the determination of the TSS of Barhi dates by using the juice obtained by pressing 100 g of destoned dates in triplicates [31].

#### 3.4.2. Determination of Firmness

The method described by Alhamdan et al. [31] was used for measuring the firmness of Barhi dates by using a TA-HDi textural analyzer model HD3128 (Stable Micro Systems, Surrey, UK). In brief, the compression of Barhi dates was done using a cylindrical probe at a velocity and a depth of 1.5 mm/s and 5 mm, respectively. The maximum power necessary to compress Barhi dates was used to construct the force–time deformation curves, which were used to calculate the firmness.

#### 3.4.3. Determination of Surface Color

The CIE surface color attributes (L; lightness to darkness, a; redness to greenness, and b; yellowness to blueness) of Barhi dates (*n* = 25 fruits) were recorded using a Hunter Lab-scan XE colorimeter (Hunter Lab, Reston, VA, USA) after calibration of the colorimeter with white plate. Ultrasound-treated Barhi dates at the first day of storage were considered as controls (positive control). The calculation of total color change (ΔE) was done using the following equation [32].

(10) $$\Delta E=\sqrt{{\left(\Delta L\right)}^{2}+{\left(\Delta a\right)}^{2}+{\left(\Delta b\right)}^{2}}\;\;\;\;\;$$

#### 3.4.4. Determination of Total Viable Count (TVC)

The determination of TVC of Barhi dates was done as described in the standard methods [33]. Briefly, Barhi dates (25 fruits) were manually destoned and then subjected to 2 min homogenization with 225 mL of sterile saline solution (0.85% NaCl) using a Stomacher^®^ 400 circulator (Seward GmbH, West Sussex, UK). Then, serial dilutions were produced by adding 1 mL homogenate to 9 mL sterile saline solution, and then, at appropriate dilution, 1 mL sample homogenate was pour plated onto a CM0309 nutrient agar media (Oxoid, Basingstoke, Hampshire, UK). After incubation at 35 °C for 1 to 2 days, the colonies in each plate were counted and results were expressed as log CFU/g sample.

#### 3.4.5. Determination of Total Phenolic Content (TPC) and DPPH Antiradical Activity

For the determination of TPC and DPPH (1,1-diphenyl-2-picrylhydrazyl) antiradical activity of Barhi dates, water extract was initially made by homogenizing 1 g of debited fruits pulp with 100 mL of ddH2O followed by sonication (Branson 2800 CPX ultrasound, St. Louis, MO, USA) for 30 min at 40 °C temperature, 40 kHz frequency, and 110 W constant power. After that, the extract was filtered using Whatman No. 1 filter paper and kept at −20 °C until used for analysis of TPC and DPPH antiradical activity. The colorimetric methods using Folin–Ciocalteu (FC) reagent and DPPH methanolic solution were used for the determination of TPC and DPPH antiradical activity of Barhi date extract as described previously [15]. For TPC analysis, 0.1 mL of Barhi date extract was mixed with 0.2 mL of 10 fold diluted FC for 5 min and then 0.5 mL of sodium carbonate (1 M) was added to the blend, methodically mixed, and allowed to stand for 2 h at room temperature. Thereafter, the absorbance was recorded at 751 nm and outcomes were specified as mg gallic acid equivalent GAE/g. For DPPH antiradical activity assay, 0.1 mL Barhi date extract was mixed with 0.2 mL of 0.25M DPPH in methanol and the mixture was preserved for 10 min in the dark at room temperature. Blank was treated in similar manner using ddH2O instead of the extract and the absorbance on the sample, and blank was carried out at 517 nm, and DPPH antiradical activity was calculated using the following equation.

(11) $$DPPH\;antiracical\;activity\left(\%\right)=\frac{A_{control}-A_{sample}}{A_{control}}$$

#### 3.4.6. Determination of Glucose and Fructose

The glucose and fructose concentration in Barhi dates were determined following the previously reported method [34] by using a Supelcosil LC-NH2 column (25 cm × 4.6 mm × 5 μm) attached to a Shimadzu HPLC system (LC10 AD, Shimadzu Corporation, Kyoto, Japan). Initially, Barhi date extract was prepared by homogenization of 5 g destoned date pulp with 100 mL ddH2O followed by 30 min incubation at 50 °C and filtration using Whatman No. 1 filter paper and, subsequently, by 0.45 μm membrane filter (Millipore, Burlington, MA, USA). After that, 20 μL was introduced to the HPLC column at 30 °C and elution of the glucose and fructose peaks were done at a flow rate of 1 mL/min using a mixture of acetonitrile and water (75%: 25%, *v*/*v*) and detected on a RID-10A refractive index detector (Shimadzu Corporation, Kyoto, Japan). The detection of glucose and fructose was obtained by comparing their retention time with that authentic standard of these sugars treated and eluted in the same way of the samples. The known concentrations of glucose and fructose were used to generate standard curves linear equations, which are used to calculate the quantity of glucose and fructose in the samples.

### 3.5. Statistical Analysis

In all treatments, triplicate samples were used and the data of physicochemical, microbial, and bioactive quality attributes obtained from them were collected and statistically analyzed with the aid of a version 18.0 SPSS software (SPSS Inc., Chicago, IL, USA). Design Expert software version 11.0 (Stat-Ease Inc., Minneapolis, MN, USA) was applied for analyzing the data of response surface methodology. Analysis of variance (ANOVA) was used to assess the effects of treatment variables (ultrasound (US) intensity, US exposure duration, and storage temperature, and duration) on the responses (glucose, fructose, TPC, TSS, firmness, ∆E, DPPH, and TVC). The statistical outcomes of adequacy precision and coefficient indicators (CV, R2, and adjusted R2) were applied to validate the adequacy and precision of the RSM models and significance was accepted at *p* < 0.05, *p* < 0.01, and *p* < 0.001 levels.

## 4. Conclusions

Barhi date fruits are commonly consumed at the Khalal maturity stage, at which the fruits have bright yellowish color, sweet taste, crunchy texture, and contain high moisture content and water activity and, therefore, have very short shelf life and required special postharvest handling and processing treatments. The present research was conducted to optimize ultrasound treatment using response surface methodology (RSM) model for extending the shelf life of Barhi dates while maintaining the physicochemical quality attributes of the fruits at different storage conditions. The results revealed that the ultrasound treatment conditions (ultrasound intensity and exposure time) and storing conditions (temperature and time) exhibited positive and negative effects on the physical, microbial, and nutritional quality characteristics of Barhi dates. The RSM model and experimental data demonstrated that the optimum ultrasound treatment for elongating the shelf life and keeping the physical, microbial, and nutritional quality features of Barhi dates were 140 W/cm^2^ ultrasound intensity, 5.2 min ultrasound exposure time, 20.9 °C storage temperature, and 21 days storage duration. Overall, ultrasound treatment could be successfully used as a postharvest processing method for improving the overall quality attributes of Barhi dates, which could be stored for 21 days at 20 °C without adverse effects on the quality attributes of these important fruits.

## Figures and Tables

**Table 1:** Ultrasound Regression coefficients for process variables and product responses.

Factors	TSS	Hardness	ΔE	TVC	TPC	DPPH	Fructose	Glucose
***Intercept***								
**β0**	27.677 *	332.997 **	156.733 ***	32.134 **	4.962 *	63.455 **	14.580 *	24.782 **
***Linear***								
**X1 (β1)**	0.677 *	−7.010 **	−8.115 ***	−1.258 **	10.360	0.660 **	1.232	1.728
**X2 (β2)**	−2.717	25.638	33.753	3.148	−31.550	−1.259	−5.101 *	−5.826 *
**X3 (β3)**	−0.988	1.9432	5.038 **	1.381 *	−25.005 **	0.693 **	−1.634 *	−2.312 *
**X4 (β4)**	−2.513 **	18.583 ***	27.525 **	4.476 *	−34.684 **	−1.611 **	−4.345	−5.322
***Interaction***								
**X1X2 (β12)**	0.016 *	0.049	−0.042	0.015 *	−0.013	−0.027	−0.029	−0.091
**X1X3 (β13)**	0.010	−0.100 *	0.058 **	0.040	0.012	−0.028 **	−0.071	−0.012
**X1X4 (β14)**	0.020 **	0.024	0.063 **	0.012 **	−0.021	0.012	0.077	0.014
**X2X3 (β23)**	0.055	0.085 *	−0.154 **	−0.024 ***	0.017	0.125	−0.013	−0.091
**X2X4 (β24)**	0.084	−0.029	0.079 **	0.011 **	−0.075	−0.025	0.012	0.072
**X3X4 (β34)**	−0.018	0.040	0.075 **	0.031	−0.044	−0.011	0.038	1.091
***Quadratic***								
**X1² (β11)**	−0.029	0.028	0.033 *	0.050	−0.041 **	−0.030	−0.049	−0.068
**X2² (β22)**	0.100	−1.083	−1.228	−0.129	1.248 **	0.023	0.208	0.242
**X3² (β33)**	0.039	−0.069	−0.179 *	−0.043	0.619 **	−0.072	0.075	0.104
**X4² (β44)**	0.092	−1.134	−1.254	−0.209 *	1.256 **	0.061	0.195	0.244
**Model F-value**	3.55	15.59	29.57	9.47	5.76	10.42	2.74	5.69
***p*** **-value**	0.019	0.001	0.0002	0.005	0.019	0.004	0.016	0.010
**Mean**	37.97	99.71	35.88	2.28	31.90	60.14	12.21	21.24
**C.V. %**	3.61	2.516	1.877	1.779	3.593	1.901	1.088	3.731
**Adeq. precision**	9.341	11.489	16.535	8.654	13.370	10.506	15.250	7.913
**R²**	0.934	0.984	0.992	0.974	0.958	0.977	0.926	0.925
**Adjusted R^2^**	0.871	0.921	0.958	0.871	0.921	0.883	0.882	0.875
**Std. Dev.**	1.37	15.12	6.73	1.32	5.08	5.42	2.55	3.68
**F-value (Lack of Fit)**	2.38	4.19	2.92	0.230	8.11	1.37	0.981	5.129
***p*** **-value (Lack of Fit)**	0.075	0.096	0.148	0.652	0.086	0.295	0.062	0.064

* *p* < 0.05, ** *p* < 0.01, *** *p* < 0.001.

**Table 2:** Independent variables and their level used for central composite design.

Independent Variables	Level				
US Intensity, W/cm^2^ (X1)	50 (−1)	100 (−0.333)	150 (0.333)	200 (1)	
US time, min (X2)	5 (−1)	10 (−0.333)	15 (0.333)	20 (1)	
Storage temperature, °C (X3)	1 (−1)	5 (−0.667)	15 (0.167)	25 (1)	
Storage time, days (X4)	1 (−1)	6 (−0.5)	11 (0)	16 (0.5)	21 (1)

## Data Availability

Not applicable.
